# Community based reproductive health interventions for young married couples in resource-constrained settings: a systematic review

**DOI:** 10.1186/s12889-015-2352-7

**Published:** 2015-10-09

**Authors:** Archana Sarkar, Venkatraman Chandra-Mouli, Kushal Jain, Jagannath Behera, Surendra Kumar Mishra, Sunil Mehra

**Affiliations:** Monitoring, Evaluation & Research Division, MAMTA Health Institute for Mother & Child, B-5, Greater Kailash Enclave-II, New Delhi, 110048 India; Department of Reproductive Health and Research, World Health Organization, Geneva, Switzerland

**Keywords:** Reproductive health, Young married couple, Community intervention, Contraception, Antenatal and postnatal care, Abortion

## Abstract

**Background:**

Most pregnancies among adolescent girls and young women aged 15–24 years occur in low- and middle-income countries (LMICs), and do so within marriage. The mortality rates and pregnancy-related morbidities are significantly higher among the women of younger age group in many South Asian and Sub-Saharan African countries. This paper presents a review of the available evidence on the effectiveness of community-based health interventions to improve the reproductive health status of young married couples in LMICs.

**Method:**

We carried out a systematic review of research studies and evaluation reports of different community-level initiatives in improving access to contraception, pregnancy care and safe abortion services by young married couples, where women were in the age-group of 15–24 years.

**Results:**

Of the 14 projects, which met inclusion criteria, eight met the quality criteria and were included in the review (five from India, two from Nepal and one from Malawi). Our analysis shows that community-based interventions consisting of counseling of young married women, and their husbands, family and community members, as well as capacity building of health workers were some of the effective measures in increasing contraceptive use, delaying pregnancy and improving pregnancy care. Stratifying young women in line with their specific reproductive health needs (newly married woman, pregnant woman, mother of one/more children) was found to be a successful innovative strategy. None of these projects explicitly addressed improving access to safe abortion care.

**Conclusion:**

Our review suggests that multi-layered community-based interventions, targeting young married women, their families and the health system can improve utilization of reproductive health services among young couples in resource-constrained settings. There is less focus on strategies to delay first pregnancy as compared to spacing among young women. Further, family and community level barriers in most of the project settings restricted its effective implementation. The paper emphasizes the need for further research to fill the knowledge gaps that exist about improving utilization of reproductive healthcare services, especially safe abortion care among young married women in LMICs.

## Background

Globally around 18 million women under the age of 20 give birth every year, representing up to one-fifth of all births, with almost 95 % of them occurring in developing countries [1]. Significant evidence show that closely spaced pregnancies (less than 18 months interval) and pregnancy before 20 years of age have strong correlation with poor maternal and neonatal health outcomes including higher rates of pregnancy related complications. In many Asian, African and Latin American countries, majority of adolescents either are married or are cohabiting before the age of 18 that adds to the problem of high maternal and neonatal morbidity and mortality [2].

In general, the reproductive health programs have traditionally focused on adult women that during recent years have expanded to include a focus on unmarried adolescents, and inadvertently neglected married young women despite their enormous need [3, 4]. The young married couples, in lower- and middle-income countries, face barriers in accessing quality reproductive health services because they are either overlooked by policies or are not reached by programs [5–7]. Their age, lack of education, limited social agency, power imbalance and inadequate negotiation skills in their marital relationships and their economic dependence trap them in a cycle of poverty with rapid and repeat childbearing [8].

While a substantial proportion of pregnancy, child bearing and parenthood occurs in women aged 15–24 years within the context of marriage or cohabitation [9, 10], they are less knowledgeable about reproductive health, and are less likely to use contraceptives and other maternal health services in comparison to women aged 25–29 years [11]. Further, young married women of many poor communities are less likely to obtain contraceptive services because they are expected to bear children soon after marriage, and being hindered in seeking antenatal and delivery care because of practical or social restrictions. Mostly, pregnancies among young married women are unplanned or poorly timed, contributing to a high rate of unsafe abortions. In 2008, an estimated 8.7 million unsafe abortions, representing 41 % of all unsafe abortions in developing regions, took place among women aged 15–24 years [12]. Thus, there is a need for evidence-based knowledge on effective ways of reaching out to young married women with the required health interventions.

This paper aims to identify and analyze effective community based interventions for improving the reproductive health outcomes of young married women (aged 15–24 years) that can be delivered in a resource-constrained setting. Community based service delivery is now widely recognized as an important strategy to deliver key maternal and child survival interventions. Interventions delivered at the community level have not only been advocated to improve access and coverage of essential services, but also to reduce the existing disparities and reaching the hard to reach populations [13, 14]. This paper systematically reviews the effectiveness of interventions delivering maternal health services to young married women that include antenatal care, delivery care, postnatal care, contraception and safe abortion.

## Methods

### Search strategy

We developed a search strategy [15] to identify peer-reviewed publications and reports of research studies and project evaluations in which interventions were delivered by community workers to improve maternal health outcomes through the increased use of contraception, safe abortion services and pregnancy care (antenatal, birth and post-natal) by young married couples in resource-poor settings. We constructed the search strategy to identify relevant articles in the following databases: PubMed, Popline, MEDLINE, JSTOR, Cochrane databases, LILACS, IMSEAR, as well as the regional databases of country specific websites of Ministry/Department of Health, and World Health Organization. We defined search strategies and search terms for each individual database through a review of the list of controlled vocabularies for databases, and constructed a stepwise approach using both indexing (MeSH) and text terms (see Table 1). Additionally, we used search engines such as Google and Google Scholar and also searched manually for relevant papers and reports. In some cases, we contacted the authors for further information including those, whose studies were finally included in this review paper.

**Table 1:** Development of search strategy

PubMed Search Strategy
Limits Activated: English, Adolescent: 15–18 years, Young Adult: 19–24 years
#1: (((Community Health Aides [MeSH]) OR (“Aide, Community Health”) OR (“Aides, Community Health Aide”) OR (“Health Aide, Community”) OR (“Health Aides, Community”) OR (“Village Health Worker”) OR (“Health Worker, Village”) OR (“Health Workers, Village”) OR (“Village Health Workers”) OR (“Worker, Village Health”) OR (“Workers, Village Health”) OR (“Family Planning Personnel”) OR (“Personnel, Family Planning”) OR (“Planning Personnel, Family”) OR (“Family Planning Personnel Characteristics”) OR (“Barefoot Doctors”) OR (“Barefoot Doctor”) OR (“Doctor, Barefoot”) OR (“Doctors, Barefoot”) OR (“Community Workers”) OR (“Community Worker”) OR (“Worker, Community”) OR (“Workers, Community”))
#2: (((Community Health Services [MeSH]) OR (“Services, Community Health”) OR (“Health Services, Community”) OR (“Community Health Service”) OR (“Health Service, Community”) OR (“Service, Community Health”) OR (“Community Health Care”) OR (“Care, Community Health”) OR (“Health Care, Community”) OR (“Community Healthcare”) OR (“Community HealthCare’s”) OR (“Healthcare, Community”) OR (“HealthCare’s, Community”)))))))))
#3: #1 OR #2
#4 (((“prenatal care” [MeSH]) OR (“prenatal care”) OR (“antenatal care”)))
#5 (((“Delivery, Obstetric” [MeSH]) OR (“Deliveries, Obstetric”) OR (“Obstetric Deliveries”) OR (“Obstetric Delivery”)))
#6 (((“Postnatal Care” [MeSH]) OR (“Care, Postnatal”) OR (“Postpartum Programs”) OR (“Postpartum Program”) OR (“Program, Postpartum”) OR (“Programs, Postpartum”) OR (“Postpartum Care”) OR (“Care, Postpartum”) OR (“Cares, Postpartum”) OR (“Postpartum Cares”)))
#7: (Contraception [MeSH]) OR (“Contraceptive Methods”) OR (“Contraceptive Method”) OR (“Fertility Control”) OR (“Female Contraception”) OR (“Contraception, Female”) OR (“Contraception, Female”) OR (“Female Contraception”) OR (“Male Contraception”) OR (“Contraception, Male”) OR (“Contraception, Male”) OR (“Male Contraception”) OR (Family Planning Services [MeSH]) OR (“Family Planning Service”) OR (“Planning Service, Family”) OR (“Planning Services, Family”) OR (“Service, Family Planning”) OR (“Services, Family Planning”) OR (“Family Planning”) OR (“Pregnancy, Planned”) OR (“Planned Pregnancies”) OR (“Pregnancies, Planned”) OR (“Planned Pregnancy”) OR (“Family Planning Programs”) OR (“Family Planning Program”) OR (“Program, Family Planning”) OR (“Programs, Family Planning”))))
#8: (((((Reproductive Behavior [MeSH]) OR (“Behavior, Reproductive”) OR (“Voluntary Childlessness”) OR (“Childlessness, Voluntary”) OR (“Delayed Childbearing”) OR (“Childbearing, Delayed”) OR (Birth Intervals [MeSH]) OR (“Birth Interval”) OR (“Birth Spacing”) OR (“Birth Spacing’s”) OR (“Pregnancy Intervals”) OR (“Pregnancy Interval”) OR (“First Birth Intervals”) OR (“First Birth Interval”) OR (Abortion, Induced [MeSH]) OR (“Induced Abortion”) OR (“Abortions, Induced”) OR (“Induced Abortions”) OR (“Abortion (Induced)”) OR (“Abortions (Induced)”)
#9: (Intrauterine Devices [MeSH]) OR (“Device, Intrauterine”) OR (“Devices, Intrauterine”) OR (“Intrauterine Device”) OR (“Contraceptive IUD”) OR (“IUD, Contraceptive”) OR (“IUDs, Contraceptive”) OR (“Contraceptive IUDs”) OR (“Contraceptive Devices, Intrauterine”) OR (“Contraceptive Device, Intrauterine”) OR (“Device, Intrauterine Contraceptive”) OR (“Devices, Intrauterine Contraceptive”) OR (“Intrauterine Contraceptive Device”) OR (“Postpartum Abstinence”) OR (“Abstinence, Postpartum”) OR (Contraceptives, Oral [MeSH]) OR (“Oral Contraceptives”) OR (“Oral Contraceptives, Phasic”) OR (Sex Education [MeSH]) OR (“Education, Sex”) OR (“Family Planning Instructors”) OR (Reproductive Health Services [MeSH]) OR (“Health Service, Reproductive”) OR (“Health Services, Reproductive”) OR (“Reproductive Health Service”) OR (“Service, Reproductive Health”) OR (“Services, Reproductive Health”)))
#10: #4 OR #5 OR #6 OR #7 OR # 8 OR # 9
#11: #3 AND #10

### Inclusion criteria

We used publications and reports, if only they fulfilled the following criteria:

- *Population:* (i) Interventions focused on young married couples, defined as married or cohabiting couples in which the female partner was in the age group of 15–24 years. (ii) Studies and evaluations of initiatives carried out in resource-constrained settings, being classified as low and middle-income countries (LMICs) by the World Bank in August 2011.
- *Intervention:* The interventions were on: (i) pregnancy care (antenatal, birth and postnatal), contraception/family planning and abortion care, and (ii) delivered by community/frontline workers, volunteers, paramedics and health workers.
- *Outcome:* Measured outcomes included changes in knowledge, attitudes, skills and practices on contraceptive use, the use of safe abortion services and pregnancy care including antenatal care (ANC), delivery care and postnatal care (PNC), and health impacts in terms of reduction in maternal mortality and morbidity.
- *Study Design:* Studies and evaluations using experimental, quasi-experimental designs, pre-post design and controlled comparison of before and after studies.
- *Language:* Studies and reports, published in English.

#### Data collection process

Our search resulted in 20,333 papers. As a first step, we reviewed titles and abstracts to identify 839 papers/project reports that appeared to meet our inclusion criteria. At the second step, we reviewed the full texts of the 839 papers and identified 14 eligible research studies and evaluations. Two authors independently assessed the papers against the inclusion criteria to determine whether those studies should be included in the review. Any difference of opinion was resolved though discussion and consultation with other authors.

**Fig. 1:**
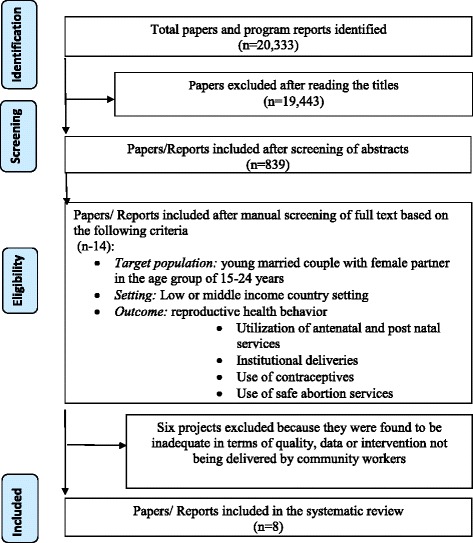
Flow diagram of systematic search results

Two authors extracted the data using a standard data extraction form. Data extracted from each article/report included country of operation, study design, target population (young married couples with a focus on women in the age group of 15–24 years), settings, components of intervention and domains of reproductive health outcomes, key findings and study limitations. In the final step, we assessed the adequacy and quality of information in those papers and reports on the study design, sample size of target population, sampling methods, interventions, evaluation methods and results. Finally, we selected eight studies/evaluations for detailed analysis (Fig. 1). As the population, intervention, methodology and outcome of these studies were heterogeneous; we decided that a meta-analysis or quantitative synthesis would not be possible to do.

### Quality assessment

We assessed the quality of methodology of each individual project. This quality assessment is similar to the pattern followed by Mirza and Jenkins checklist of first eight items [16]. The following criteria included to assess the quality of study: (i) explicit study objectives, (ii) adequate sample size, (iii) representative sample, (iv) clear inclusion and exclusion criteria, (v) measures of reproductive health- reliable and valid, (vi) response rate reported and losses explained (vii) adequate description of data, and (viii) appropriate statistical analyses.

## Results

As stated above, from the selected list of fourteen studies/project reports, six were excluded because they lacked adequate information for review or had interventions, which were not being delivered through community health workers/frontline functionaries [17–22], or had inadequate data for review [20–22]. Eight project reports [23–30] met the inclusion criteria for the systematic review. We have assigned acronyms for easy reference: FP (Promoting Healthy Timing and Spacing of Births in India through a Community-based Approach- Frontiers Program) [23], FTP (First Time Parents) [24], PRACHAR (Promoting Change in the Reproductive Behavior of Youth) [25], ACQUIRE (Mobilizing Married Youth in Nepal to Improve Reproductive Health: The Reproductive Health for Married Adolescent Couples project) [26], REWARD (Reaching and Enabling Women to act on Reproductive Health Decision) [27], MMM (Encouraging Contraceptive Uptake by Motivating Men to Communicate about Family Planning: The Malawi Male Motivator Project) [28], KEM (Reproductive and Sexual Health Education, Care and Counseling for Married Adolescents In Rural Maharashtra) [29] and FRHS (Social Mobilization or Government Services: What influences Married Adolescents’ Reproductive Health in Rural Maharashtra, India) [30] (Table 2).

**Table 2:** Program intervention of different projects with name of the implementing organization

Abbreviations of different projects	Topic	Organization
FP [23]	Promoting healthy timing and spacing of births in India through a community-based Approach	Population council, Lala Lajpat Rai Memorial Medical College, and (Department of Economics) Jamia Milia Islamia University
FTP [24]	Empowering married young women and improving their sexual and reproductive health: Effect of the First-time Parents Project	Population Council, CINI and Deepak Charitable Trust
PRACHAR [25]	The effect of community-based reproductive health communication interventions on contraceptive use among young married couples in Bihar, India	PATHFINDER INTERNATIONAL.
ACQUIRE [26]	Mobilizing married youth in Nepal to improve reproductive health: The Reproductive Health for Married Adolescent Couples Project, Nepal, 2005-2007	ACQUIRE Project partners Engender Health and CARE
REWARD [27]	Determining an effective and Replicable communication-based mechanism for improving young couples’ access to and use of reproductive health information and services in Nepal-An Operations Research Study	Center for Research on Environment, Health and Population Activities
MMM [28]	Encouraging contraceptive uptake by motivating men to communicate about family planning: The Malawi Male Motivator Project	Family Health International and Save the children
KEM [29]	Reproductive and sexual health education, care and counseling for married adolescents in rural Maharashtra	KEM Hospital Research Centre (KEM), Pune
FRHS [30]	Social mobilization or Government services: What influences married adolescents’ reproductive health in rural Maharashtra, India?	Foundation for Research in Health Systems, Maharashtra

The Table 2 provides the full project titles and details of the implementing organizations of the included projects. Two studies [25, 28] were described in both peer-reviewed papers and unpublished reports, whereas the remaining six were unpublished project reports. The identification details, methodology, intervention components and outcomes of the included studies are summarized in Table 3. The effectiveness of the included interventions in improving the reproductive health outcomes are presented in Tables 4. The appraisal of quality is presented in Table 5.

**Table 3:** Methodological details of the projects included in the systematic review to improve reproductive health choices for young married couples in resource-constrained setting through public health system

Reference/country/implementation period	Objectives	Target population	Multi component intervention			Study design and sampling technique, evaluation design	Focus of intervention
			Direct intervention with young married couple	Interventions to target family members and community meetings	Interventions towards health and other welfare systems		
Khan et al. 2008, [23] India, 2006–07 (FP)^a^	1. To assess the feasibility and effectiveness of using community workers to promote the use of Lactation Amenorrhea Method (LAM) and post-partum contraception that are conducive to healthy spacing and timing of pregnancy which can lead towards maternal and child mortality control.	1. Pregnant women with parity 0, 1 Age group-20-24 years	1. Individual counselling	1. Discussion sessions,	1. Cascaded capacity building training of frontline health workers	1. Quasi experimental with intervention/control area,	1. Post-partum contraception,
		2. Husbands of pregnant women	2. Group counselling	2. Educational campaign		2. Purposive and convenience sampling,	2. Family planning
		3. Mother-in-Law of pregnant women	3. Wall Paintings			3. Comparison between intervention and control	
Santhya et al. 2008, [24] India, 2003–05, (FTP)^a^	1. To develop and test an integrated package of health and social interventions to improve married young women’s reproductive and sexual health knowledge and practices,	1. Young women married for up to 2 years and not yet pregnant	1. Individual counselling	1. Informal interaction to provide information	1. None	1. Quasi experimental with intervention/control area,	1. Antenatal care,
	2. To enhance their ability to act in their own interest and expand their social support networks.	2. Nulliparous pregnant women;	2. Group Formation	2. Community Educational campaign		2. Selection process not reported	2. Delivery care
		3. First-time mothers up to 18 months postpartum	3. Group counselling			3. Comparison between intervention and control, and Baseline end line	3. Post natal care
							4. Contraceptive use to delay first pregnancy
							5. Decision making, mobility and couple communication
Daniel et al.2008, [25] India 2005–07 (PRACHAR)^a^	1. To improve the health and welfare of young mothers and their children by changing traditional customs of early childbearing	1. Married women (aged 15–24)	1. Individual counseling	1. Community meetings	1. Training of rural medical practitioner (RMP)	1. Quasi experimental with intervention/control area,	1. Delay first birth
		2. Unmarried adolescents	2. Street Plays,	2. BCC		2. Purposive and convenience sampling, Comparison between baseline and follow-up with in intervention and control group	2. Family planning
		3. Young couples, their guardians (parents and in laws) and influential community members	3. Drama	3. Group meeting		3. Follow-up/monitoring of cohort for reproductive and sexual health	3. RTI/STI prevention,
		4. Subgroups based on child status (no child, pregnant and with one child)	4. Community educational campaign,				4. Importance of delaying first birth, need of birth spacing,
			5. Provision of basic reproductive and sexual health services				
ACQUIRE^b^ 2008, Nepal, [26] 2000–2002 (ACQUIRE Project)^a^	1. To increase married adolescents’ knowledge about family planning, maternal health and HIV and STI,	1. Married couples in which the woman was an adolescent	1. Group training	1. Advocacy workshop	1. Advocacy	1. Pre post Repeated Cross sectional surveys	1. Use of contraception before first pregnancy,
	2. To increase community and family support for reproductive health decision making by married adolescent couples, especially related to family planning and pregnancy, delivery and post natal care	2. Youth couples aged up to 25 years	2. Individual counselling	2. Discussion sessions,		2. Cluster Sampling with Village Development Council as unit, Comparison between Baseline and End line	2. ANC,
			3. Sensitization			3. Qualitative and quantitative survey	3. Delivery,
			4. Community educational campaign				4. PNC
			5. Street Plays				5. HIV/AIDS,
			6. Drama				6. Gender attitude
CREHPA^b^ 2004, Nepal, [27] (REWARD^a^ Project)	1. To improve the reproductive health need of newly married couples	1. Young married women under 25 years	1. Group formation Youth Communication Action Group (YCAG) and Mothers group (MG)	1. Special events	1. Cascaded Capacity building training of frontline health workers	1. Quasi experimental with intervention/control area,	1. Family planning,
			2. Capacity building of Group leaders and deputy leaders, Group (YCAG and MG)	2. Dissemination of knowledge	2. Advocacy	2. Purposive and convenience sampling,	2. ANC,
			3. Counselling, Linkage of Group (YCAG and MG) members to health providers	3. Sensitisation		3. Comparison between intervention and control groups	3. Delivery
			4. Community Educational campaign			4. Qualitative and quantitative survey	4 PNC,
			5. Group Sensitization				5. STI and HIV/AIDS
			6. Street Plays, Drama, Wall Paintings, Radio channels, TV channels				
Shattuck et al. 2011, [28] Malawi, 2008 (MMM)^a^	1. To evaluate the effect of a peer-delivered educational intervention, the Malawi Male Motivator intervention, on couples’ contraceptive uptake	1. Primary target: Men at least 18 years old and married to or living with female sexual partner aged not less than 25 years who was not currently pregnant or breastfeeding	1. Information, Motivation and Behavioral skill model	1. None	1. None	1. RCT	1. Use of contraception
		2. Secondary target group: Wife/female sexual partner of those men				2. Computer based random number list, Comparison between baseline and end line with in intervention and control group	2. Gender norms
						3. Qualitative and quantitative survey	
Pande et al. (a) 2006 [29] (KEM)^a^	1. Improving the reproductive health of married and unmarried adolescents	1. Married male and female adolescents and young adults aged: 14–25 years	1. Identification and referral for counselling	1. Sensitisation	1. Training	1. Feasibility study, House-listing survey,	1. Sexual and Reproductive health knowledge and status and use of services
			2. Couple counselling,			2. Baseline and end line comparison	
			3. Marital counselling, and Clinical Referral			3. Qualitative and quantitative survey	
Pande et al. (b) 2006 [30] (FRHS)^a^	1. To examine the feasibility and effectiveness of providing a package of services in a rural community to improve married adolescents’ sexual and reproductive health knowledge and status, and use of services	1. Newly married couple less than 22 years old,	1. Group Formation and Group counselling (health education sessions),	1. Social mobilization activities	1. Training of health providers	1. 2 × 2 intervention control design	1. ANC
		2. Husbands	2. Couple counselling			2. House-listing survey	2. Delivery,
		3. Mothers in law.				3. Comparison between baseline and end line with in intervention and control group	3. PNC
							4. Contraception use,
							5. Abortion, infertility and treatment of reproductive RTI

^a^Abbreviations commonly used for these projects

^b^Institutional evaluation (no author listed)

**Table 4:** Effectiveness of interventions included in the systematic review on reproductive health outcomes of young married women

Projects	ANC	Institutional delivery	PNC	Contraception
Khan et al. 2008 (FP) [23]^c^				1. Proportions discussing about spacing and family planning (%)
				% of couples who discussed family planning methods: Exp. Group (Exp.): 61 % (*N* = 560); Con. Group (Con): 39 % (*N* = 570); Sig (*p* < = 0.001)
				% of couples who discussed when to have next child: Exp.: 85 %, con: 85 %; Non Sig *Z test, Exp.* vs. *Con.*
				2. Contraceptive use at 9 months post-partum
				% of women currently using family planning (FP): Exp: 63 %, Con: 32 %; Sig (*p* < = 0.001)
				% currently pregnant: Exp.: 10 %, Con: 16 %; Sig (*p* < = 0.001) *Z test, Exp.* vs. *Con.*
				*(Data Source: End line Survey)*
Santhya et al. 2008 (FTP) [24]	Comprehensive antenatal care received by first time mothers:	% of first time mothers who reported institutional delivery	First time mothers who reported receiving routine checkups within six weeks postpartum	1. Use of contraceptive to delay first birth:
	Diamond Harbour (DH):	Diamond Harbour (DH):	Diamond Harbour (DH):	Diamond Harbour (DH) site:
	Con; BL: 6.5^a^; EL: 7.7 (*N* = 244,500); Sig.	Con; BL: 61 %; EL: 70 % (*N* = 244,500); Sig	Con.; BL: 7 %; EL: 27 % (*N* = 244,500); Sig	Con.; Baseline (BL): 54 %; End-line (EL): 66 % (*N* = 212, 238),
	Exp. non-intervention;	Exp. non-intervention;	Exp. non-intervention; BL:	Exp. non-intervention^b^; BL: 24 %;
	BL: 6; EL: 6.9, (*N* = 206, 191); Sig.	BL: 43 %; EL: 49 %, (*N* = 206, 191); Non sig	6 %; EL: 15 % (*N* = 206,191); Sig	EL: 34 % (*N* = 94, 281)
	Exp. intervention; BL: 6.1; EL: 7.7 (*N* = 114,460); Non sig	Exp. intervention; BL: 40 %; EL: 51 % (*N* = 114,460); Sig	Exp. intervention; BL: 6 %; EL: 45 % (*N* = 114/460); Sig	Exp. intervention; BL: 28 %; EL: 39 % (*N* = 163, 96)
	Vadodara (VD):	Vadodara (VD):	Vadodara (VD):	Vadodara (VD) site:
	Con; BL: 6.3; EL: 7.9 (*N* = 270,314); Sig	Con; BL: 56 %; EL: 68 % (*N* = 270,314); Sig	Control; BL: 29 %; EL: 33 % (*N* = 270/314); Sig	Control; BL: 36 %; EL: 13 % (*N* = 259, 338); Sig
	Exp.non-intervention; BL: 7.1; EL: 7.8 (*N* = 228,159); Sig	Exp. non-intervention; BL: 65 %; EL: 77 % (*N* = 228,159); Sig	Exp. non-intervention; BL: 26 %; EL: 49 % (*N* = 228/159); Sig	Exp. non-intervention; BL: 34 %; EL: 11 % (*N* = 176, 310); Sig
	Exp. intervention; BL: 8.3; EL: 8.8 (*N* = 61,285); Non sig	Exp. intervention; BL: 71 %; EL: 70 % (*N* = 61,285); Non Sig	Exp. intervention; BL: 28 %; EL: 51 % (*N* = 61,285); Sig *Data Source: Baseline End line Survey*	Exp. intervention; BL: 18 %; EL: 21 %, Non Sig
Daniel et al. 2008 (PRACHAR) [25]^c^				1. % of Married women aged 15–24 years who were using contraception
				BL; Control: 2.8 %, Intervention: 4.3 %
				Follow –up period: Control: 4.7 %, Intervention: 20.7 %, Sig
				Interventions vs. Control: (OR: 3.8:1.0; *p* < 0.001)
				2. % increase in median interval (in months) between marriage and first birth:
				BL; Intervention area: 21.3 months, Non-intervention areas: NA
				EL- Intervention area: 24 months; Non-intervention areas: NA
				*(Data Source: Baseline and Follow-Up Data)*
ACQUIRE, Evaluation and Research Studies Nepal 2008 [26]	Women who attended ANC on 4 or more occasions	Decrease in proportion of home delivery:	Use of PNC increased: Use of postnatal care services by young married women	1. Actual use of contraception to postpone first birth among young married women:
	BL: 29.4 % and EL: 49.9 % (*N* = NA)	BL: 75 % and EL: 67 % (*N* = NA)	BL: 20 % and EL: 30 % (*N* = 240, 269)	BL: 4.4 % and EL: 4.8 %, (*N* = 294, 333)
	Note: NA = data on sample size is Not Available		*(Data Source: Baseline and End line Survey)*	
CREPHA, Nepal2004 (REWARD Project) [27]	Attended 4 or more ANC visits:	% of young married women who delivered their last child in hospital:		1. Contraceptive prevalence rate: (%) among young married women
	MG area: BL: 24.8 %, EL: 52.9 %; Sig	YCAG area: BL-5.9 %, EL: 16.1 %		Youth Communication Action Groups (YCAG) area; BL: 26 %, EL: 28 %
	Control area: BL: 14.4 %, EL: 43.2 %; Sig	Control: BL-2.6 %, EL: 5.3 %		
				Mothers’ Group (MG) area; BL: 19 %, EL: 37 %
		*(Data Source: Baseline and End line Survey)*		Control area; BL: 29 %, EL: 34 %
				2. Mean age at pregnancy among young married women
				YCAG area; BL: 18.1 %, EL: 18.5 %
				MG area; BL: 18.0 %, EL: 18.3 %
				Control area; BL: 17.7 %, EL: 17.5 %
Shattuck et al. 2011 (MMM) [28]^c^				Contraceptive uptake among young men with married or co-habiting partner:
				OR for contraceptive uptake among young males between intervention and control groups: OR (95 % CI): 2.4 (1.45,4.03) Sig
				OR for increase in communication frequency among young couples between intervention and control groups: OR (95 % CI): 0.61 (0.36, 1.02) Sig
				*(Data Source: Baseline and Post-Intervention Data)*
				Note: OR indicates Odds Ratio
Pande et al. (a) 2006 (KEM) [29]	Knowledge regarding Regular ANC check-ups			Awareness improved with respect to condom use as a way to prevent STIs and HIV
	Pre: 50 %, post: 75 %			Note: Data is not available
	*(Data Source: Pre and Post-Intervention Data)*			
Pande et al. (b) 2006 (FRHS) [30]	Change in knowledge among young married women on need for ANC check-ups between baseline and end line:			Knowledge on side effects of contraceptive use has increased
				Note: Data is not available
	Social Mobilization arm (SM) only: 24.2 %, Government Services arm (GS) only: 18.5 %, SM + GS: 12 %, Control: 18.9 %			
	Change in knowledge among young married women on danger signs of pregnancy between baseline and end line:			
	SM only: 24.4 %, GS only: 22.5 %, SM + GS: 24.2 %, control: 16.5 %			
	*(Data Source: Baseline and End line Survey)*			

*Exp* experimental, *Con* control, *Sig* significant, *DH* Diamond Harbor, *VD* Vadodara, *SM* social mobilization, *GS* government health services, *MG* mothers group, *YCAG* youth communication and action group, *OR* odds ratio

^a^Mean score on index value based on comprehensive antenatal care indicators

^b^Those residing in the experimental villages but did not participate in the intervention

^c^Did not include/analyze the data on pregnancy care

**Table 5:** Checklist for quality of projects included in systematic review to improve reproductive health choices for young married couples in resource constrained setting through public health system

Project	Explicit aim	Sample size justification	sample representative of population	Inclusion and exclusion criteria stated	Reliability and validity of measures justified	Response rate and drop out specified	Data adequately described	Statistical significance assessed	Discussion of generalizability	Ethical clearance	Score
Khan et al. 2008 [23]	Yes	Yes	Yes	Yes	Yes	Yes	Yes	Yes	Yes	Yes	10
Santhya et al. 2008 [24]	Yes	No	No	Yes	Yes	Yes	Yes	Yes	Yes	Yes	8
Daniel et al. 2008 [25]	Yes	No	Yes	Partly	Yes	No	No	Yes	Yes	Not reported	5
ACQUIRE 2008 [26]	Yes	Partly	Yes	No	No	No	Yes	Yes	Yes	Not reported	5
CREHPA Nepal 2004 [27]	Yes	Yes	Yes	No	Yes	Partly	Yes	Yes	Yes	Yes	8
Shattuck et al. 2011 [28]	Yes	No	No	Yes	Yes	No	Yes	No	No	Yes	5
Pande et al. (a) 2006 [29]	Yes	No	No	Yes	Yes	No	No	No	No	Not reported	2
Pande et al. (b) 2006 [30]	Yes	No	No	Yes	No	No	No	No	No	Not reported	2

*No, Not reported, Partly* were given 0 score whereas *Yes* was given 1 score

### A: Characteristics of the included projects

i) ***Project Area and Population:*** Out of the eight studies/project reports included in this review, five studies/project reports were from India, two were from Nepal and one from Malawi. Of the five projects implemented in India, FTP by Santhya et al. [24] was conducted in 12 villages of two districts each in the states of Gujarat and West Bengal; whereas KEM and FRHS were implemented in Maharashtra, [29, 30] covering two blocks of a district each. FP (Khan et al. 2008) was implemented in one district of Uttar Pradesh [23]. PRACHAR (Daniel et al.) has covered 19 blocks in three districts in phase I, 10 additional blocks in phase II and III each in the state of Bihar [25]. The project area and population covered under each implementation varied widely; the largest [25] involved 19 administrative blocks of 25–35 villages each, covering a population of 2.8 million, whereas FRHS (Pande et al. b 2006) was restricted to one village covering 129 couples only in Maharashtra [30]. Of the two projects in Nepal, one was by the ACQUIRE project that covered 69 Village Development Committees with an approximate population of 502,000 [26], whereas the other one (REWARD) reached 7577 young married couples in 62 village clusters [27]. The study in Malawi (MMM) was implemented in 257 rural villages across 17 traditional authorities in Mangochi district [28]. All the projects were implemented in rural areas targeting adolescents, young married women, men and couples.
ii) ***Study Design:*** Of the eight studies/project reports reviewed, four – FP, FTP, PRACHAR and REWARD [23–25, 27] used quasi-experimental study designs with intervention and control arms. MMM [28] used a 2 × 2--randomized control design in which young men either married or having a female partner less than 25 years of age were assigned randomly. Three project reports– FTP, MMM and KEM [24, 28, 29] compared baseline and end line measures in the intervention and control arms. FRHS [30] used a feasibility approach focusing on the processes and dynamics of implementation rather than on its outcome. ACQUIRE [26] carried out pre and post cross-sectional surveys.
iii) ***Sample Characteristics:*** Sample size calculations and their bases were explained in three [23, 27, 28] of eight studies/project reports. One initiative- REWARD [27] used a two-stage cluster sample design while FP used random allocation of individuals or clusters [23]. The number of participants in the baseline, mid-intervention and end line samples were not mentioned by the KEM [29] projects. In the PRACHAR project [25] socio-demographic characteristics between intervention and control populations were matched. In other projects, this was not done. The sample selection process was not mentioned in the project reports [24, 25, 29, 30].
iv) **Project Goals:** The overall objectives of all projects included improving knowledge, skills (accessing social support and independent decision-making), and service uptake for reproductive health in terms of maternal healthcare and using contraception for delaying/spacing pregnancies among young married women/adolescents. Six out of eight projects: FTP, PRACHAR, ACQUIRE, REWARD, KEM and FRHS [24–27, 29, 30] focused on adolescents/young married couples’ reproductive and sexual health knowledge and practices, whereas FP developed and tested a model to educate young mothers about birth spacing and increased use of lactational amenorrhea and post-partum contraception. One initiative- PRACHAR [25] focused on educating parents and influential family members of unmarried adolescents regarding sexual health issues, including the demerits of early child bearing and marriage. All studies/project reports, except two– REWARD and MMM [27, 28] built the capacity of service providers to improve quality of services to meet the specific needs of young married couples. Four of the studies/project reports– ACQUIRE, REWARD, KEM and FRHS [26, 27, 29, 30] delivered a package of services for improving sexual and reproductive health (SRH), sexually transmitted infections (STI) and reproductive tract infections (RTI) testing among rural married adolescents. However, abortion was not explicitly focused in any one of these studies/projects.

### B: Components of intervention

All the studies/project reports, included in the review, have employed multi-component interventions targeting young married couples, and/or their family members, and/or the community and health systems. The list of intervention components is presented in Table 3.

i. ***Direct interventions with young married women/couples:*** In almost all studies/project reports, the direct intervention with young married women focused on individual counseling during home visits. The young married women in most of the studies/project reports were in the age group of 15–24 years, although two projects– PRACHAR and ACQUIRE [25, 26] targeted younger women in the age group of 10–24 years. In all studies/project reports [23–27, 29, 30] apart from MMM [28], the major strategy for direct intervention included formation of groups of young married women in the community to expand their social support network and enhance their ability to act in their own interests; group counseling (information on reproductive and sexual health issues by peer educator/frontline functionary/change agent/project staff); and direct information provision to the young married women/couples through home visits.
  Three studies/project reports – FP, REWARD and KEM [23, 27, 29] used couple counseling, where couples were visited and counseled separately in their homes. Two studies/project reports– PRACHAR and ACQUIRE [25, 26] used street plays, drama and wall paintings while FTP [24] created safety net through small savings for use in emergencies. Two projects FP and PRACHAR [23, 25] used Community Educational Campaigns to promote healthy timing and spacing of pregnancy among young couples. Two projects had specific tailor made interventions for young women in different life stages [24, 25]. Daniel et al. stratified young married couples in different groups like newly married couples, who had not yet had a child received information about delaying and spacing pregnancies and young couples with one child received information about spacing subsequent children [25]. Further, FTP stratified young married women as newly married, first time parent and first time mother [24]. Most of the projects used locally available educational materials such as pocket booklets, poster-sized flex charts and flip charts for disseminating reproductive health information.
ii. ***Interventions to target family members and community:*** The interventions with family and community attempted to influence the decision makers and gatekeepers of the primary target group of young married couples. In-laws (mothers, fathers and sisters), community leaders, community elders and other members were the main target groups in all the studies/project reports apart from MMM [28]. Most projects used group counseling through community meetings, and opportunistic interactions on health and economic benefits of delaying pregnancy, prevention of RTI/STI and improving reproductive healthcare by involving husbands. Two studies/project reports– ACQUIRE and REWARD [26, 27] used the local media to disseminate information for creating a supportive environment. Social mobilization activities were carried out in the community through community groups by FP, PRACHAR and KEM [23, 25, 29] and FTP and ACQUIRE [24, 26] celebrated special days. There was no quantitative evidence obtained for the effect of intervention on change in attitude and behavior of family members and/or at community level.
iii. ***Interventions targeting health and other systems:*** Sensitization of government health system/health workers at Primary Health Center (PHC) level on reproductive and sexual health issues of young married women/adolescents were done in three studies/project reports [26, 29, 30]. FP and FRHS [23, 30] involved doctors/clinical psychologists from the public health system. Three studies/project reports– FP, FTP and REWARD [23, 24, 27] used cascaded capacity building activities through training frontline functionaries like Accredited Social Health Activist [ASHA], Anganwadi Worker [AWW] and Auxiliary Nurse Midwife [ANM] and three community-based change agents (peer educator, schoolteacher and local resident) [25, 26, 30]. Traditional birth attendants and rural health practitioners were trained in two projects- FTP and ACQUIRE [24, 26] and rural medical practitioners in PRACHAR [25]. Media channels were used in PRACHAR only. However, there was no quantitative evidence on how much change is effected by health system strengthening for providing the young married women/adolescents better access to reproductive healthcare services at local level.

### C: Behavioural outcomes of the included projects

The key behavioral outcomes of the review included contraception use, delaying of first pregnancy, antenatal care, delivery and postnatal care and abortion services among young married women.

i) ***Contraceptive use:*** Overall 6 out of 8 studies/project reports – FP, FTP, PRACHAR, ACQUIRE, REWARD and MMM [23–28] focused on improving knowledge and use of contraception among young couples. Knowledge on contraception use increased significantly following the intervention in PRACHAR [25] (Control vs. Intervention, OR- 1.0:3.8, *p* < 0.001) and FP [23] (Control: (39 %) vs. Experimental (61 %), *p* < 0.001) whereas in REWARD, there was a slight increase that was not significant [27]. There were improvements in the attitudes of young couples towards contraceptive use in three studies/project reports – PRACHAR, REWARD and MMM [25, 27, 28]. In PRACHAR, there was a statistically significant change [Follow-up: (Comparison {72 %} vs. Intervention {80 %}, *p* < 0.01) among married women (15–24 years), who agreed that contraceptive use is safe and necessary for delaying first birth [25]. In four projects – FP, PRACHAR, REWARD and MMM, [23, 25, 27, 28] increase in contraceptive use among young married women was found, with statistically significant increase in two of them- FP (Control vs. Experimental, OR- 1.0: 1.63, *p* < 0.001) and MMM (Control vs. Experimental, OR- 1.0: 2.4, *p* < 0.05) [23, 28]. Increased spousal communication regarding spacing and family planning was observed in FP, whereas discussion with mother-in-law/daughter-in-law regarding timing of second pregnancy did not show any significant improvement [23]. Further, decline in household violence and improved mobility was observed in FP and FTP [23, 24]. Overall, findings highlight that contraceptive use was improved by 1–18 % in different project areas.
ii) ***Delay in first pregnancy:*** Four studies/project reports– FP, FTP, PRACHAR and ACQUIRE [23–26] included postponing the age of first pregnancy as one of their objectives. Only one of them – FTP [24] showed a statistically significant difference in delaying first pregnancy between intervention and control arms (Vadodara: Control vs. Intervention, OR- 1.0: 1.96, *p* < 0.001). Another one- PRACHAR [25] showed a delay of 3 months at first pregnancy from baseline to end line, but the increase was not statistically significant. Persuading young couples to delay their first birth was found to be more difficult in comparison to convincing them for birth spacing and use of family planning [24].
iii) ***Use of antenatal care (ANC):*** Five studies/project reports – FTP, ACQUIRE, REWARD, KEM and FRHS [24, 26, 27, 29, 30] aimed to improve knowledge and practices of ANC (reported receiving antenatal checkup within first trimester, had comprehensive antenatal check-up including urine test, blood pressure measurement, ultrasonography, advice on home based practices including three nutritious meals and adequate rest). In three of these – FTP, ACQUIRE and REWARD [24, 26, 27] showed improvements in ANC service utilization. The number of ANC checkups received by first time mothers increased substantially from baseline to end line in FTP [24] (in Diamond Harbor, Baseline: 33 %, End line: 53 %), ACQUIRE [20] (Baseline: 29 %, End line: 50 %) and REWARD (Mothers Groups: Baseline: 20 %, End line: 54 %) [27]. The change in antenatal care access is ranged from 1–28 % in different project areas.
iv) ***Delivery (intra-natal) care:*** Only three out of eight studies/project reports: FTP, ACQUIRE and REWARD [24, 26, 27] focused on improving birth preparedness among young married women. One initiative—FTP [24] showed an improvement in birth preparedness; however, there was no statistically significant increase in institutional delivery in both the intervention groups in Vadodara (Gujarat) and Diamond Harbor (West Bengal). In the REWARD [27] project, there was a slight increase in the awareness of at least three danger signs during labor in end line (1.6–8.5 %), but overall levels remained low. Further in this study, levels of institutional delivery declined in one of the intervention groups from baseline to end line (i.e. in others group 2.8–1.1 %). In another project – ACQUIRE, while the proportion of couples, who discussed where to give birth increased significantly (Baseline: 24 %, End line: 40 %), whereas the rates of institutional delivery did not increase significantly (Baseline: 24 %, End line: 31 %) [26]. On an average, the access to institutional delivery had raised by about 10 % in intervention areas.
v) ***Postnatal care:*** Only three studies/project reports: FTP, ACQUIRE and REWARD [24, 26, 27] focused on improving postnatal care among young married women. Two of them – FTP and ACQUIRE [24, 26] showed a significant improvement in the proportion of first-time mothers receiving routine checkups within 6 weeks of postpartum. The range of effects of different intervention projects on postnatal care were varied from 10–39 %.
vi) ***Abortion Services:*** Only one initiative KEM [29] included abortion as an outcome. In this study, only one of the intervention arms (i.e. the social mobilization group) showed an increase in knowledge on safe abortion.

### D. Program barriers

The programs faced several barriers in implementation due to different social and cultural barriers. Orthodox gender norms that accord women low social status, limit their education and severely restrict their mobility were still widespread in project areas. This often led to lower participation in the intervention/group sessions and often required a lot of effort by community health workers to persuade them and their families. In FP [23], where involvement of mother-in law was a key objective, more than 50 % of young women could not share IEC booklets of the program with their mothers-in law due to social distance and hesitation for discussing issues related to reproductive health and timing of pregnancy. Further, frequent movements of pregnant women to their marital and natal homes diminished the exposure to intervention for many participants and led to “recall biases.” Moreover, misconceptions regarding, use of contraception before first pregnancy leading to infertility was commonly reported in the project areas [26].

There were also barriers observed at the health system level. Married adolescents did not access services due to lack of confidentiality, privacy and poor staffing at health facilities. Training and orientation of health service providers often took longer time than expected, thus delaying implementation of different service components. In a few projects, some of the intervention components were initiated once the need has become apparent, rather than from the start e.g. in KEM [29] where, couples as community educators were initiated through mid-way, thus had limited scope to make generalization.

## Discussion

**Fig. 2:**
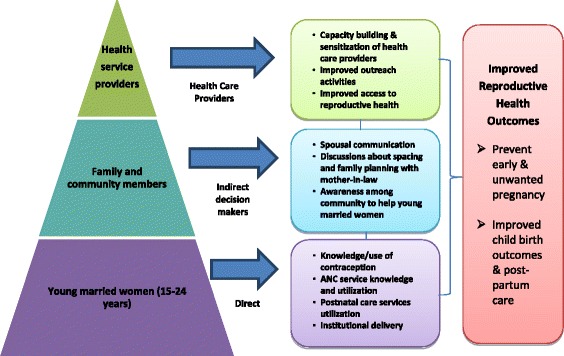
Schematic representation of multi-layered intervention to improve reproductive health access among young married women

Our systematic review showed that a combination of community-based interventions targeting young married couples, influential family members, community members and health systems were effective in delaying pregnancy, increasing contraceptive use and pregnancy care (Fig. 2). The interventions, which were shown to be effective in reaching young married couples to educate and motivate them for positive healthcare seeking behaviors include formation of women groups, involving them in group-counseling sessions, home visits by frontline functionaries/outreach workers [23, 24, 26, 27, 30] and support them with the establishment of a small fund (village health fund) for use in emergencies [24]. Interventions, shown to be effective in addressing family and community members included group counseling [23–27] and opportunistic interactions [24] with them. The latter included street plays [26, 27], wall paintings [23], involving community groups and local media [27] to spread the message. Further, the strategy of stratifying young women in line with specific needs of women in different reproductive life stages (newly married, nulliparous pregnant women, couples with one child and pregnant women with one/more children) was found to be effective in reaching them with the required package of interventions [24, 25]. Interventions to make health workers and health facilities or systems more responsive included sensitizing managers and training different cadres of health workers active in the community, in providing education, counseling and health services [25, 30]. Training of health service providers, paramedics and community workers on the health-service needs of young married women/couples improved the knowledge of the target group on reproductive health as well as service utilization for contraception and pregnancy care [23, 26, 27].

There is substantial evidence that community based interventions have the potential to improve maternal and newborn health outcomes. A recent review of literature on community level interventions to improve quality of care for maternal health indicated that home visits, community mobilization and training of community health workers and traditional birth attendants have the maximum potential to improve reproductive health outcomes [31]. The determinants of reproductive health behaviors including health care comprehensive package of intervention is expected to improve reproductive health access of young married women.

Although there is very little evidence on the role of community based interventions in improving the reproductive health of young married couples [15–24 years], two reviews on young women’s contraception use highlight the importance of community-based multifaceted interventions [32, 33]. Consistent to our findings, a recent literature review on reaching first time parents for healthy spacing of second and subsequent pregnancies found that programs that successfully combine a number of approaches to offer an integrated package of information and appropriate services for a woman, her partner, her support network and her access to health services will likely to be most effective in increasing reproductive decision making, use of contraceptives and better spacing of additional pregnancies [32]. Further, another systematic review on contraceptive use among young women (married and unmarried girls in the age group of 11–24 years) indicated that community wide, multifaceted interventions and the combined provision of information, life skills, support and access to youth friendly health services are necessary to reduce the barriers that restrict young women’s contraceptive choices across developing countries [33].

The review highlighted the importance of addressing influential family members as well as young people that was reiterated by the findings of FTP [24]. It showed significant change in indicators such as use of contraception to delay the first birth, uptake of comprehensive antenatal care; delivery preparation, routine postpartum check-ups and breastfeeding practices, but it excluded institutional delivery at first birth. The reason being many young women had gone to their natal homes for first delivery; and the interventions they were exposed to, were not being able to influence family or health service environments outside the project sites. Our review showed that the key determinants of health and healthcare seeking behavior need to be understood and then can be addressed using a combination of tailor-made approaches. This was entirely in line with the recommendations of two documents–one published by WHO [2] and the other by UNFPA [34]; although the focus of both documents was beyond healthcare seeking for contraception, abortion and maternal health.

However, the findings of our review need to be interpreted in terms of paucity of data on community based interventions on young married couples, as only eight studies/project reports met our inclusion criteria. Most of the programs tend to focus on young/adolescent (i.e. youth friendly health services especially for unmarried youth), married women or mothers in general. There are a couple of interventions that focus on the specific information and service delivery needs of young married women with a child. There are far less interventions on delaying first pregnancy than on spacing or delaying second and subsequent births among young married women.

Our search was only limited to English language publications. Another important limitation was that all projects included in this review were from South Asia except one. Further, the studies/project reports had some methodological problems including inadequate information about fidelity of implementation [25, 29], sample selection [24, 25, 29, 30], and methods to control confounding and/or contamination [24, 26, 27]. In addition, there were disparities between the proposed objectives of some studies/project reports and the outcomes assessed after intervention, thus making it impossible to know, if they have really achieved their objectives. For example, two of them [24, 26] set out to increase the knowledge and capacity of healthcare providers to meet the needs of married young women/adolescents; however, at the end line neither of these studies provided any data on these objectives. One of the projects [27] aimed to increase contraceptive negotiation skills of young married women, but it did not provide any evidence about the outcome of this objective at the end line.

Most of the projects had reported loss to follow up of the target women/couples in the study, which might have led to participation bias. One project reported contamination of intervention effects in the control arm [23], leading to difficulty in identifying changes attributable to the intervention. High baseline values and proximity to the control area were a potential source of bias in one project [24]. Both the projects from Nepal were interrupted by political conflicts, unrest and closure of health facilities for long durations, and so the effectiveness had to be interpreted cautiously. Finally, none of the studies/project reports have evaluated changes in health outcomes (maternal and neonatal morbidity/mortality) and none of them addressed abortion care explicitly.

## Conclusion

Our review suggests that community based interventions targeting young married couples, their immediate family, community members and health service providers contribute positively to improving access and utilization of reproductive health services in resource-constrained settings of low and middle-income countries. The projects were mainly restricted to low income countries in South Asia and Africa, and had limitations in the methodology and evaluation design. The primary outcomes evaluated were contraceptive use, delaying first pregnancy, use of antenatal care, delivery care, post-natal care and abortion services. Further, none of the projects included maternal or neonatal mortality and/or morbidity as well as abortion as one of their health outcomes.

By adopting these intervention strategies, the national reproductive health programs could help reducing unwanted pregnancies, pregnancy-related morbidity and mortality in adolescents and young people in other similar settings. Beside this, there is a pressing need for more research in this field, considering that a large proportion of pregnancy occurs within marriage at a young age in low and middle-income countries. Future efforts should be made to include collection of robust data in well-designed studies to generate evidence on costs and outcomes for improving reproductive health services for young married women.

### Ethical statement

An ethical statement was not required for this work.

## Abbreviations

ANC: Antenatal Care
PNC: Post natal Care
SRH: Sexual and Reproductive Health
STI: Sexually Transmitted Infections
RTI: Reproductive Tract Infections
BCC: Behavior Change Communication
IEC: Information Education Communication
ASHA: Accredited Social Health Activist
ANM: Auxiliary Nurse Midwife
AWW: Anganwadi Worker
PHC: Primary Health Center
